# Anthropogenic impact on diazotrophic diversity in the mangrove rhizosphere revealed by *nifH* pyrosequencing

**DOI:** 10.3389/fmicb.2015.01172

**Published:** 2015-10-21

**Authors:** Hongmei Jing, Xiaomin Xia, Hongbin Liu, Zhi Zhou, Chen Wu, Sanjay Nagarajan

**Affiliations:** ^1^Sanya Institute of Deep-sea Science and Engineering, Chinese Academy of SciencesSanya, China; ^2^Division of Life Science, The Hong Kong University of Science and TechnologyKowloon, Hong Kong; ^3^Department of Civil and Environmental Engineering, Faculty of Engineering, National University of SingaporeSingapore, Singapore

**Keywords:** Diazotrophs, *nifH*, Pyrosequencing, Mangrove

## Abstract

Diazotrophs in the mangrove rhizosphere play a major role in providing new nitrogen to the mangrove ecosystem and their composition and activity are strongly influenced by anthropogenic activity and ecological conditions. In this study, the diversity of the diazotroph communities in the rhizosphere sediment of five tropical mangrove sites with different levels of pollution along the north and south coastline of Singapore were studied by pyrosequencing of the *nifH* gene. Bioinformatics analysis revealed that in all the studied locations, the diazotroph communities comprised mainly of members of the diazotrophic cluster I and cluster III. The detected cluster III diazotrophs, which were composed entirely of sulfate-reducing bacteria, were more abundant in the less polluted locations. The metabolic capacities of these diazotrophs indicate the potential for bioremediation and resiliency of the ecosystem to anthropogenic impact. In heavily polluted locations, the diazotrophic community structures were markedly different and the diversity of species was significantly reduced when compared with those in a pristine location. This, together with the increased abundance of *Marinobacterium*, which is a bioindicator of pollution, suggests that anthropogenic activity has a negative impact on the genetic diversity of diazotrophs in the mangrove rhizosphere.

## Introduction

Mangrove ecosystems constitute a large portion (i.e., ∼60–70%) of the coastline in the tropical and subtropical regions, where they play an essential role in maintaining the sea level and protecting the coast (Duke et al., 2007). Mangroves are highly productive ecosystems but in general they are nitrogen deficient. Their high productivity might be partially attributed to the high rate of biological nitrogen-fixing activity via diazotrophs in sediments and in the rhizosphere of mangrove trees, which contributes ∼40–60% of the total nitrogen required by the ecosystem (Holguin et al., 2001). Diazotrophs are therefore responsible for most of the nitrogen input in the mangrove ecosystem (Holguin et al., 2001). When comparing the nitrogen-fixing activity of the mangrove rhizosphere to that in sediments (Zuberer and Silver, 1978; Sengupta and Chaudhuri, 1991), it is the former that contributes more significantly (by supplying most of the nitrogen requirements) to the health and sustenance of mangrove ecosystem (Gotto and Taylor, 1976; Zuberer and Silver, 1978; Holguin et al., 1992, 2001).

Nitrogen-fixing bacteria and archaea fix atmospheric dinitrogen (N_2_) via the nitrogenase protein complex (Howard and Rees, 1996), which is encoded partially by the *nifH* gene. This gene has been used to classify diazotrophs into Clusters I-IV (Chien and Zinder, 1996) and is thus suggested to be a suitable marker for the phylogeny of diazotrophs. Nitrogen-fixing bacteria, such as *Azospirillum*, *Azotobacter*, *Rhizobium*, *Clostridium*, *Klebsiella*, *Vibrio*, and *Phyllobacterium* sp. have been isolated from the rhizosphere of various mangrove species (Sengupta and Chaudhuri, 1991; Holguin et al., 1992; Cevallos et al., 1996). However, laboratory isolates usually poorly represent the microbial populations living in the natural environment. Therefore, molecular techniques, such as denaturing gradient gel electrophoresis (DGGE) and sequencing of *nifH* gene clone libraries, have been applied to environmental samples to study the diversity of the diazotroph communities in the mangrove rhizospheres (Flores-Mireles et al., 2007; Rameshkumar and Nair, 2009; Liu et al., 2012) and sediment samples (Zhang et al., 2008; Dias et al., 2012; Romero et al., 2012; Sun et al., 2012). In recent years, pyrosequencing, which is a next generation sequencing approach that provides a more comprehensive perspective of the microbial community, has been employed to study the bacterial (dos Santos et al., 2011) and diazotrophic community structures (Gomes et al., 2010) in mangroves. However, until now, the application of high-throughput pyrosequencing to study the functional *nifH* gene in diazotrophs living in the mangrove rhizosphere has not been demonstrated.

**FIGURE 1:**
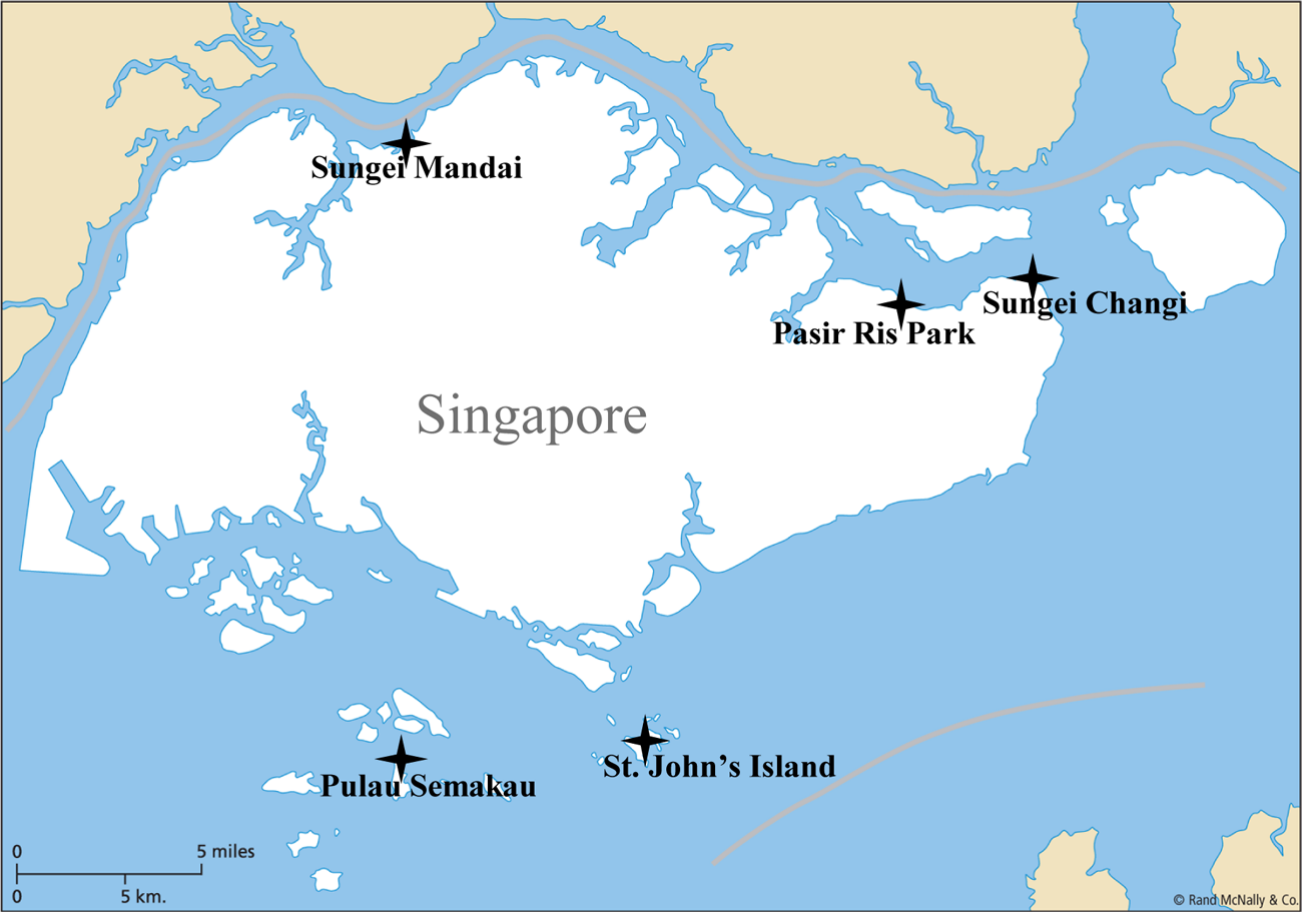
**The five mangrove sampling stations located along the Singapore coastline**.

In recent years, mangroves have been highly threatened by both natural and anthropogenic disturbances, with a disappearance rate of ∼1–2% per year across their range (Duke et al., 2007). The increased input of external nutrients into mangrove sediments from adjacent areas might cause significant variations in the composition and activity of the nitrogen fixers. No specific associations have been found between the mangrove tree species and the nitrogen-fixing bacteria that were isolated. Instead, fertilizer and organic amendments, such as oil (Dias et al., 2012) and polycyclic aromatic hydrocarbons (Sun et al., 2012), along with the bioavailability of nutrients (Romero et al., 2012, 2015), have caused significant alterations in the diazotroph communities in different mangrove ecosystems. Rhizosphere diazotrophs are not only affected by root–bacteria interactions, but are also driven by the geochemical parameters in sediments (Zhang et al., 2008; Romero et al., 2012). In order to better understand the anthropogenic and ecological impact on the diazotrophic community structure in the mangrove rhizosphere, samples were collected from five tropical mangroves along the north and south coastline of Singapore, namely Sungei Mandai (SM), Pulau Semakau (PS), Sungei Changi (SC), Pasir Ris Park (PRP) and St. John’s Island (SJ) (**Figure 1**). Among them, SM is located to the northwest and downstream of Lim Chu Kang, which is characterized by strong agriculture activities. PRP was chosen because in December 2009, the first major outbreak of toxic algal bloom in Singapore coastal waters occurred near the maritime space of this mangrove, and as a result, thousands of farm fish were killed. This mangrove still had the highest total nitrogen content during our sampling in 2012. SC is located in the northwestern region of Singapore near Changi airport and downstream of PRP and Sungei Punggol, the latter being an old landfill, which was closed in 1999 and is now a wetland reserve. PS and SJ are both located along the northern coastline. The former is a new landfill in Singapore, whereas the latter is located far from any industrial and residential areas and is thus influenced the least by human activities. SJ was therefore used as a pristine mangrove area in our study. The influence of anthropogenic perturbation on the rhizospheric diazotrophs in the tropical mangrove was investigated using high-throughput 454-pyrosequencing of the functional *nifH* gene in order to provide new insights into the impact of environmental stress on the mangrove microbial communities.

## Materials and Methods

### Sample Collection and DNA extraction

Triplicate mud samples were collected from mangrove rhizosphere in five locations: PRP; PS; SC; SJ; and SM, along the coastline of Singapore in October 2012 (**Figure 1**). The dominant species of the mangroves in all five sites are *Avicennia alba*. Characteristics of the five locations are described in **Table 1**. Sediments down to about 5 cm adjacent to the rhizosphere were collected after roots being removed and placed in 15 ml Falcon tubes. They were then kept on ice in the field after which they were stored at -80°C prior to further analysis.

**Table 1:** Characteristics of the five locations used in this study^§^ .

	PRP	PS	SC	SJ	SM
Locations	1°22′ N 103°57′ E	1°12′N 103°45’E	1°23′N 103°59’E	1°13′ N 103°50′E	1°26’N 103°46′E
pH	8.61 ± 0.09	8.12 ± 0.11	6.37 ± 0.23	7.10 ± 0.02	7.45 ± 0.04
Temp (°C)	27.20 ± 0.07	27.90 ± 0.57	–	28.30 ± 0.07	27.10 ± 0.14
^∗^Conductivity (μs/cm)	2,712 ± 70	2,676 ± 277	2,007 ± 90	2,626 ± 243	1,021 ± 37
Moisture (%)	24.26 ± 0.18	22.39 ± 0.68	16.18 ± 0.37	22.18 ± 4.16	37.33 ± 0.99
NO^3-^ (μg/g)	58.03 ± 0.12	55.53 ± 1.68	50.56 ± 1.85	46.61 ± 1.50	64.29 ± 3.92
NO^2-^ (μg/g)	2.42 ± 0.02	2.54 ± 0.05	2.55 ± 0.18	2.41 ± 0.08	2.32 ± 0.17
NH_4_^+^ (μg/g)	1.26 ± 0.01	1.67 ± 0.39	0.41 ± 0.03	0.31 ± 0.12	0.95 ± 0.24
TP (μg/g)	8.67 ± 2.13	124.50 ± 7.60	–	5.74 ± 1.21	288.80 ± 39.2
N:P ratio	7.12	0.48	–	8.59	0.23
Cr (μg/g)	3.91 ± 0.28	6.79 ± 0.89	49.26 ± 1.37	7.20 ± 1.94	11.86 ± 1.21
Mn (μg/g)	70.65 ± 7.92	520.00 ± 66.10	73.96 ± 0.85	94.92 ± 75.31	44.60 ± 4.35
Fe (μg/g)	11,382 ± 270	10,440 ± 1011	838 ± 432	10,735 ± 2,729	27,716 ± 1,683
Co (μg/g)	4.29 ± 0.11	2.22 ± 0.18	35.19 ± 0.51	1.16 ± 0.38	0.77 ± 0.01
Ni (μg/g)	3.91 ± 0.10	4.19 ± 0.37	44.71 ± 0.44	3.61 ± 0.70	3.44 ± 0.07
Cu (μg/g)	3.64 ± 0.34	–	53.71 ± 15.45	7.99 ± 1.77	7.64 ± 0.30
Zn (μg/g)	22.01 ± 1.04	10.51 ± 0.37	85.43 ± 6.13	122.80 ± 86.40	57.14 ± 5.00
Cd (μg/g)	–	–	72.39 ± 1.36	–	0.26 ± 0.13
Ga (μg/g)	6.54 ± 0.33	15.59 ± 0.38	11.40 ± 1.04	3.23 ± 0.78	17.10 ± 2.45
Al (μg/g)	5,627 ± 712	2,707 ± 554	8,631 ± 1847	2,952 ± 814	20,569 ± 5,398
Pb (μg/g)	9.09 ± 1.02	3.95 ± 0.39	73.40 ± 6.05	11.37 ± 3.00	21.90 ± 2.24
As (μg/g)	2.37 ± 0.83	21.17 ± 1.86	36.92 ± 1.19	2.82 ± 1.90	19.96 ± 1.26
Ba (μg/g)	52.64 ± 3.77	5.06 ± 1.16	60.40 ± 1.78	10.25 ± 4.13	6.90 ± 1.19

*^§^ All data in this table have been cited from Xia et al., in preparation; ^∗^conductivity of the surface of the mangrove sediment.*

Genomic DNA from three independent samples was extracted and pooled together (∼250 mg) using the PowerSoil DNA extraction kit (Mo Bio Laboratories, Carlsbad, CA, USA) according to the manufacturer’s protocol. DNA concentrations were quantified with a NanoDrop 2000 UV-Vis Spectrophotometer (NanoDrop Products, Wilmington, DE, USA).

### Biogeochemical Analysis

The location of each sampling site was recorded via GPS (global positioning system) (**Table 1**). The *in situ* temperature and salinity were measured with a thermometer and salinometer, respectively, and the conductivity was measured using the practical salinity scale according to UNESCO (1985). The wet and dry weights of each sample were recorded before and after being heated in a conventional oven, and moisture was calculated as the ratio of weight difference over the wet weight. pH was measured with a Schott Gerate pH meter using a glass electrode, which was inserted into the pore water.

Total nitrogen in the form of NH_4_^+^, NO_3_^-^, and NO_2_^-^ was tested after the soil samples were sonicated and filtered through a 0.45 μm polytetrafluoroethylene membrane filter, and quantified with a Metrohm AG (Herisau, Switzerland) ion chromatograph (IC) equipped with a 733 IC analytical separation system (Yasuhara et al., 1999). Metals such as Pb, Cd, Zn, Fe, Mn, Cu, Cr, and Ni were measured with an inductively coupled plasma-mass spectrometer (ICP-MS, MLAN 6100, Perkin Elmer, Waltham, MA, USA) following microwave digestion (EPA3051) (Moor et al., 2001).

### Amplification and 454 Pyrosequencing

Genomic DNA samples were used as templates for amplification of the *nifH* gene of approximately 360 bp following the nested PCR protocols of Zehr et al. (1998) and using a FastStart High Fidelity PCR system, dNTPack (Roche, Switzerland) with a Peltier Thermal Cycler (Bio-Rad, USA). In order to enable sample multiplexing during sequencing, barcodes were incorporated between the adapter and forward primer. Nuclease-free water was used as the negative control in each reaction. Triplicate PCRs were performed for each sample and the amplicons were pooled and subsequently purified with the illustra^TM^GFX^TM^PCR DNA and Gel Band Purification kit (GE Healthcare, Little Chalfont, Bucks, UK). An amplicon library was constructed with equimolar concentrations of the amplicons, and emPCR was conducted according to the Rapid Library preparation kit instructions (Roche, Switzerland). DNA beads were successfully deposited onto the PicoTiterPlate and sequenced with a GS Junior system (Roche, Switzerland).

### Post-run Analysis

The *nifH* sequences generated in this study were processed using the microbial ecology community software program Mothur (Schloss et al., 2009). De-noise and removal of barcode and forward primer sequences were applied simultaneously with theshhh.flows and trim.seqs scripts, and chimeric sequences were identified with chimera.uchime. Reads of less than 250 bp in length, and sequences with undetermined nucleotides were removed. The remaining sequences were translated into amino acid sequences using RDP Framebot^1^ and sequences containing in-frame stop codon(s) were excluded.

A local BLAST was performed using the nifHrefseqs database from NCBI^2^. Sequences showing less than 80% similarity with the database sequences were removed. The remaining DNA sequences were used for operational taxonomic units (OTUs) and rarefaction analysis with 95% sequence similarity as the cutoff value (Kong et al., 2011; Santos et al., 2014). OTUs that contain one sequence were removed. The Chao1 richness estimator and diversity (Shannon–Weaver index, *H′*) were calculated with 95% sequence similarity after sequence normalization; this resulted in equal numbers of sequences for each sample by randomly selecting within each sample according to the sample with the least number of sequences. Normalized OTU data were also used for generating a Venn diagram using R (Ihaka and Gentleman, 1996). A newick-format tree describing the dissimilarity among multiple groups was generated with the tree.shared command, and the Thetayc calculator was used to determine the unweighted pair group method with arithmetic mean (UPGMA).

To identify the phylogenetic affiliation of *nifH* sequences, a neighbor-joining (NJ) tree was constructed using the molecular evolutionary genetics analysis (MEGA) software (Tamura et al., 2007) for the 20 most abundant OTUs together with selected reference sequences from different diazotrophic groups. In addition, redundancy analysis (RDA) was performed to reveal the relationships between diazotrophic assemblages and environmental variables using CANOCO V4.5 (Ter Braak and Smilauer, 2002). All data were root-square transformed and the effects of high collinearity among factors were removed. Forward selection was used to determine the minimum set of environmental variables that could explain the largest amount of variance in the microbial community. The statistical significance of an explanatory variable added in the course of forward selection was tested with the Monte Carlo permutation test (999 permutations, *p* ≤ 0.05). For all community ordination analyses, biplot scaling was used.

### Accession Number

All the *nifH* sequences obtained from this study have been deposited in the National Center for Biotechnology Information (NCBI) Sequence Read Archive (SRA) under the following accession numbers: SAMN03318353 for PRP (Pasir Ris Park); SAMN03318354 for SM (Sungei Mandai); SAMN03318355 for SC (Sungei Changi); SAMN03318356 for PS (Pulau Semakau) and SAMN03318357 for SJ (St. John’s Island).

## Results

### Characteristics of the Sampling Locations

Among the five sampling locations, the pristine SJ served as a background, whilst the other mangroves were polluted to different extents. The two northern locations, SC and SM, had a relatively lower salinity (conductivity), and SM had relatively higher concentrations of NO_3_^-^, NH_4_^+^ and total phosphorus (TP, **Table 1**). SC, which is strongly affected by the old landfill and nearby airport, exhibited the lowest pH, temperature and moisture but the highest content of heavy metals (Cr, Co, Ni, Cd, Pb, As, Ba). In contrast, PRP, PS, and SJ had undetectable levels of Cd. In addition, SM had unusually high concentrations of Al and Fe, and the lowest salinity as a result of strong agricultural activity. The highest pH was found in PRP. The two southern locations, PS and SJ, had a similar temperature and salinity, but the former had much higher nutrient levels (NO_3_^-^, NO_2_^-^, and NH_4_^+^). All sites had a relatively low nitrogen to phosphorus (N:P) ratio, ranging from 0.23 in SM and 0.48 in PS, due to high TP, to 7.12 and 8.59 in PRP and SJ, respectively.

### Sequencing Statistics and Diversity Estimates

Pyrosequencing generated more than 20,000 raw sequence reads for each sample, and a total of 53,348 reads remained (**Table 2**), after the low quality reads were filtered out according to the criteria described in Section “Materials and Methods.” On average, there were 10,670 reads per sample and an average length of 347 bp per read was obtained. Using a 5% sequence cutoff value, a total of 5,381 OTUs were obtained (**Table 2**). The northern polluted locations (i.e., SC and SM) in general had far fewer OTUs than the other locations, with the lowest number of OTUs occurring in SC. The highest number of OTUs, indicating the species richness and diversity, occurred at SJ. In addition, coverage for all the samples ranged from 92.9 to 99.8%, consistent with the pattern reflected by the rarefaction curves (Supplementary Figure S1): curves for SC and SM reached a plateau earlier than the other samples; and the curves for all the samples became saturated, suggesting that sufficient sampling efforts were applied in this study to allow adequate assessment of the microbial community composition in each sample.

**Table 2:** Sequencing statistics and diversity estimates for the samples collected from the five locations in this study.

Locations	Total reads	Average length (bp)	High quality reads	OTU (95%)	Chao1(95%)	ACE (95%)	Shannon (95%)	Coverage (95%)
PRP	20,857	349	9,678	1,296	1,374.35	1,472.23	6.065	0.953
PS	25,787	348	14,099	1,669	1,786.52	1,870.76	6.344	0.931
SC	27,348	342	6,998	128	130.56	136.75	2.593	0.998
SJ	23,830	348	12,988	1,708	1,795.78	1,942.12	6.349	0.929
SM	20,179	349	9,585	580	584.32	609.39	4.739	0.986

**FIGURE 2:**
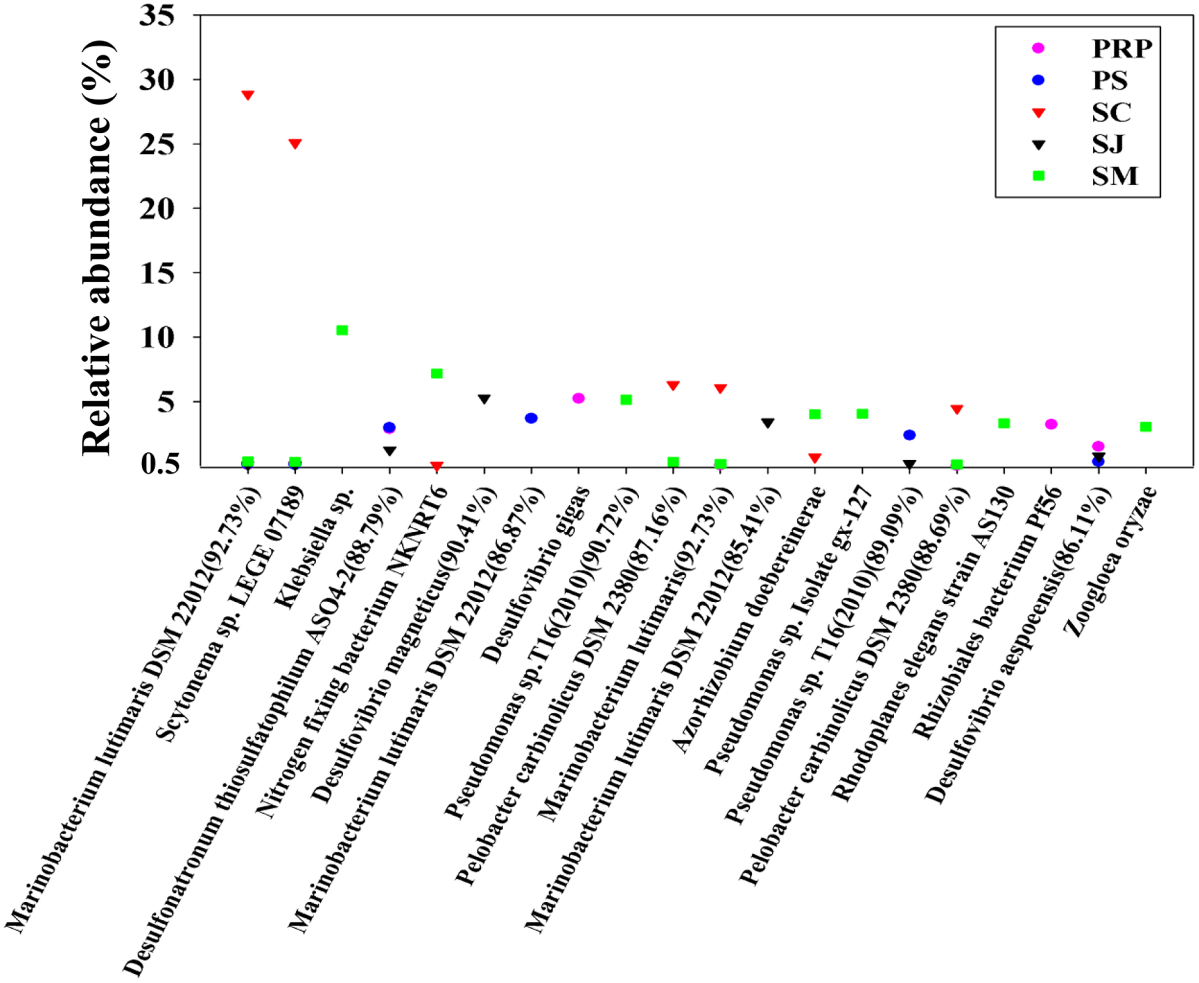
**Identity and distribution of the 20 most abundant OTUs (with 95% similarity as the cutoff value) among all samples collected from the five mangrove locations in Singapore**.

The distribution of the 20 most abundant OTUs was highly varied among the different samples with the 12 top OTUs occurring in SM. OTU1 and 2 accounted respectively for 29 and 25% of the total OTUs in SJ, and showed a high similarity with *Marinobacterium lutimaris* DSM22012 and *Scytonema* sp. LEGE07189, respectively. The abundance of the remaining OTUs was less than 6% for each, except for OTU3, which accounted for 11% in SM showing close affiliation with *Klebsiella* sp. (**Figure 2**). In addition, a BLAST search provided the identities of the 10 most abundant OTUs in each sample, and these were shown to vary considerably such that the top OTUs in SC and PS were both closely affiliated with *Marinobacterium lutimaris* DSM22012 with different levels of similarities; whereas in PRP and SJ, they were affiliated with *Desulfovibrio gigas* and *Desulfovibrio magneticus*, respectively, and in SM, they were affiliated with *Klebsiella* sp. (Supplementary Figure S2).

### Phylogeny and Community Composition of Diazotrophs

**FIGURE 3:**
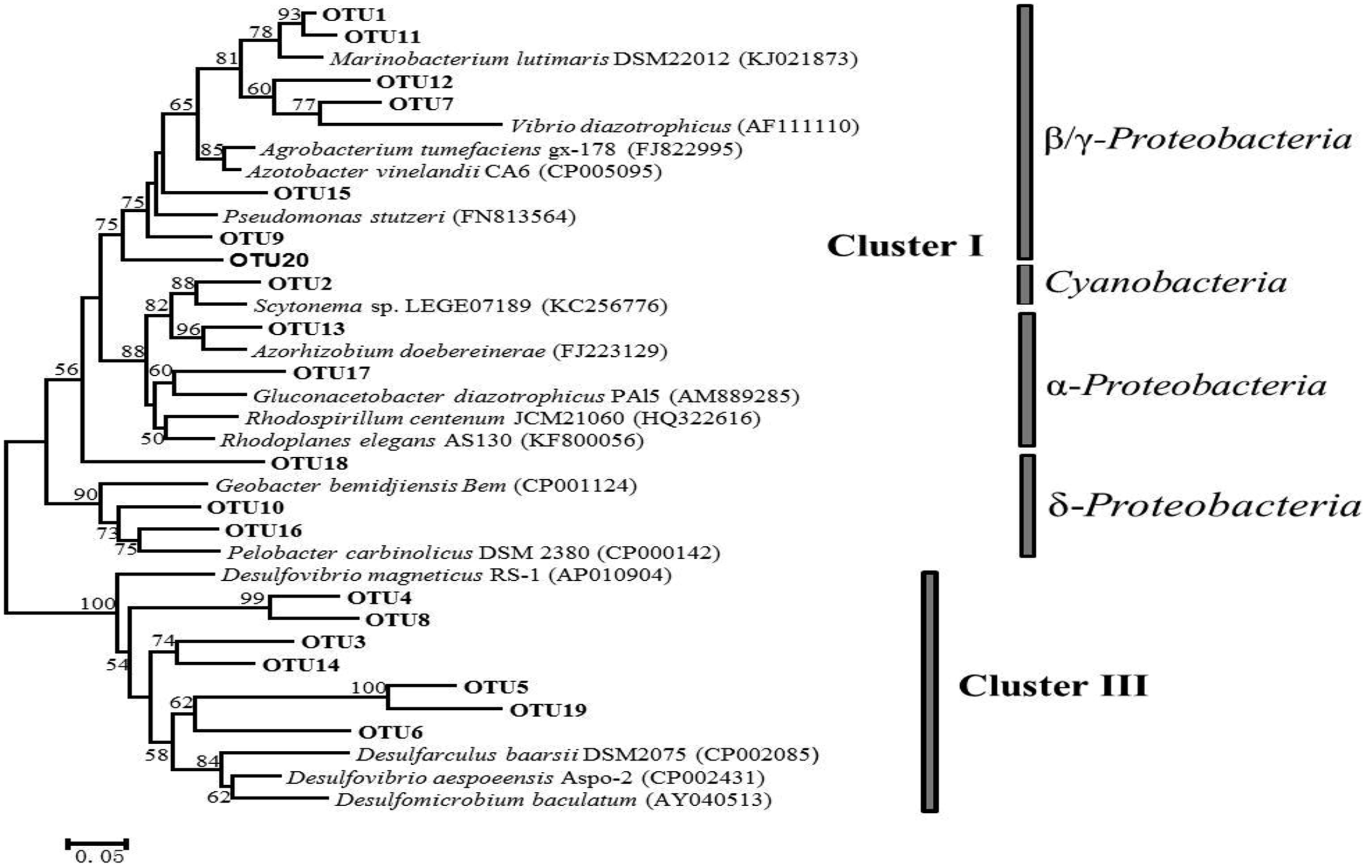
**A neighbor-joining (NJ) phylogenetic tree illustrating the 20 most abundant OTUs (with 95% similarity as the cutoff value) among all the samples collected from the five locations in Singapore.** Canonical *nifH* clusters are indicated to the right of the figure. A bootstrap value greater than 50% is shown (calculated 1,000 times).

**FIGURE 4:**
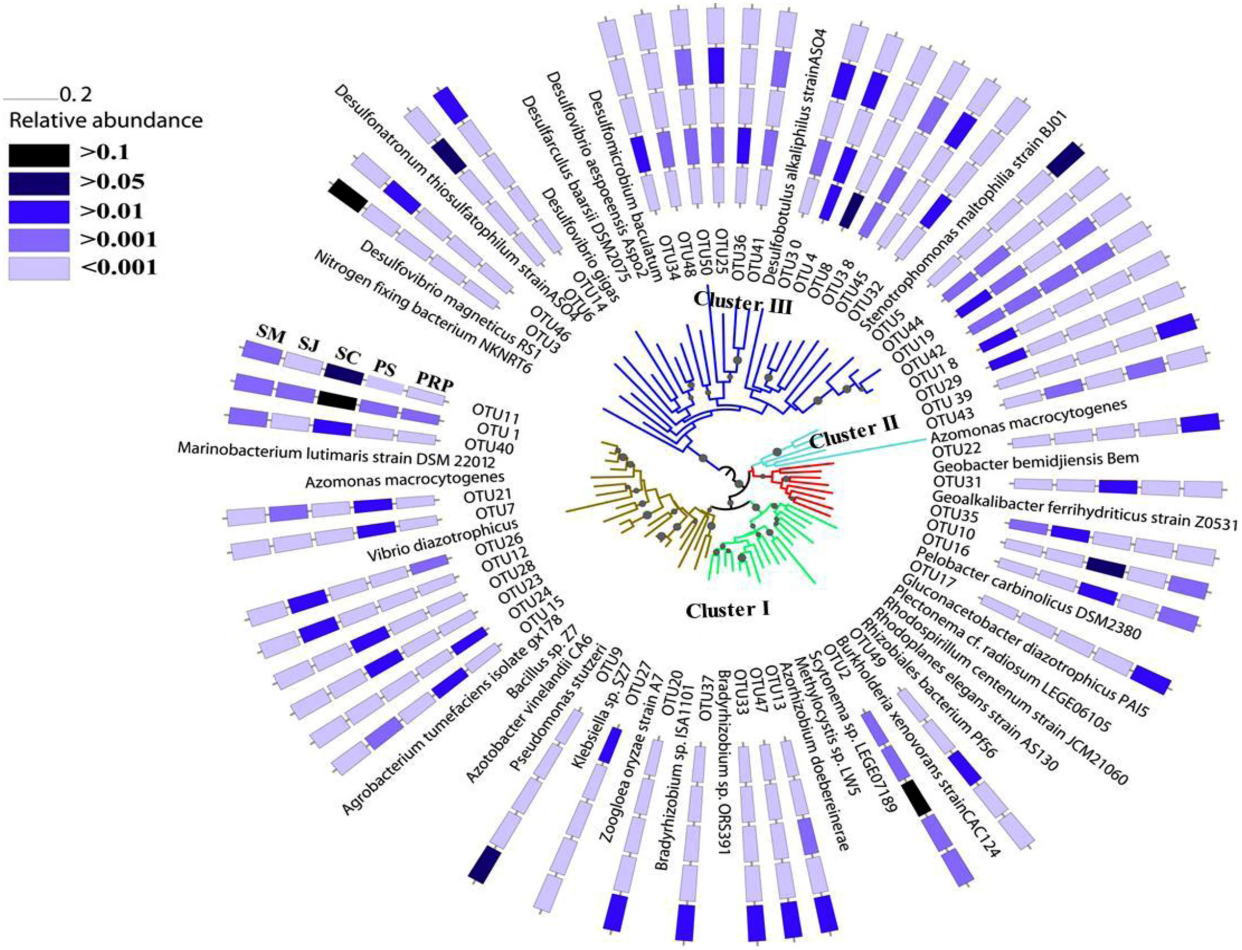
**A NJ tree to show the phylogenetic relationship of the most abundant 50 OTUs with affiliated canonical *nifH* clusters (Chien and Zinder, 1996) indicated by colored branches.** Bootstrap value greater than 50% is shown (calculated 1,000 times).

Phylogenetic trees constructed using NJ and maximum-likelihood methods had congruent tree topology. The NJ phylogenetic tree for the 20 most abundant OTUs demonstrated that 13 of them fell into cluster I, and the rest were in cluster III (**Figure 3**). Among the cluster I OTUs, seven were affiliated with *Marinobacteium lutimaris*, *Vibrio diazotrophicus*, and *Pseudomonas stutzeri*, which belong to the class of *β/γ-Proteobacteria*; one was affiliated with *Scytonema* sp., a cyanobacterial diazotroph; two were affiliated with *Azorhizobium doebereinerae* and *Gluconacetobacter diazotrophicus* in the *α-Proteobacteria* class; and two were closely related to *Pelobacter carbinolicus* in the *δ-Proteobacteria* class. Within cluster III, four OTUs grouped with *Desulfobotulus alkaliphilus*, whereas the remaining three were clustered with *Desulfovibrio* sp. In addition, a phylogenetic tree based on the 50 most abundant OTUs (**Figure 4**) indicated that several (i.e., OTU18, 29, 39, and 43) fell into Cluster II, and were affiliated with *Azomonas macrocytogenes*.

**FIGURE 5:**
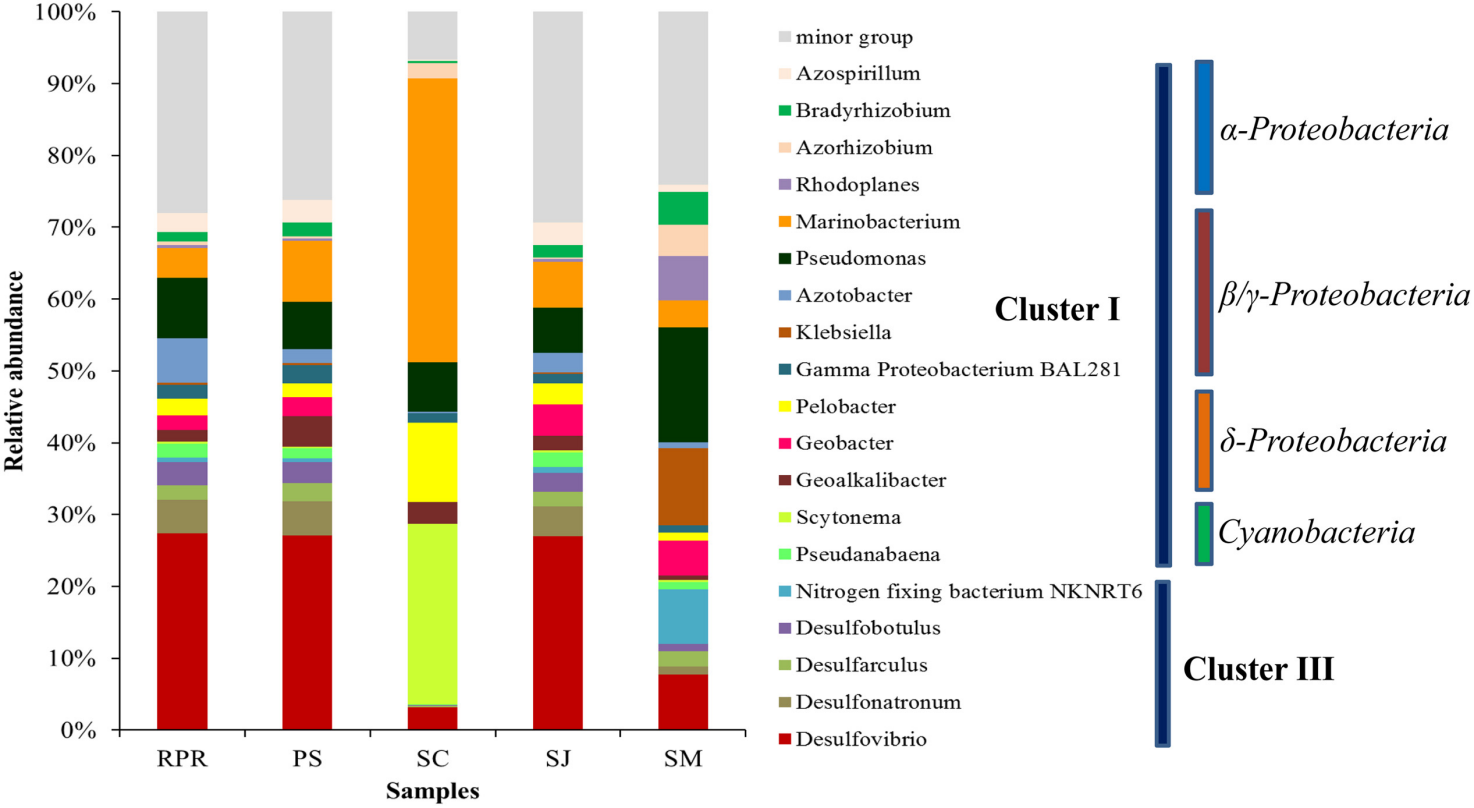
**Community composition of diazotrophs at the genus level for samples collected from the five locations in Singapore.** Phylogenetic groups accounting for less than 2% of the total community were treated together as minor group.

In total, 19 major diazotrophic groups at the genus level were identified from all the samples (**Figure 5**). Groups each accounting for less than 2% of the total community were grouped together and defined as minor groups. Genera *Desulfarculus*, *Desulfobotulus*, *Desulfonatronum, Desulfovibrio* and nitrogen-fixing bacterium NKNRT6 belong to Cluster III, whereas the remaining 14 genera belong to Cluster I. Samples from SC and SM contained more Cluster I than Cluster III groups, and the predominance of Cluster I (89.9%) was more apparent in SC with only 0.3% of Cluster III found. Cluster III was slightly more abundant than Cluster I in samples from PRP, PS and SJ. Minor groups accounted for more than 20% in all samples, except for SC (<10%).

Within the Cluster I diazotrophs, the β*/*γ*-Proteobacteria* class was the most abundant in all the samples especially in those from SC and SM, where *Marinobacterium* and *Pseudomonas*, respectively, showed the highest relative abundance (**Figure 5**). The cyanobacterial diazotrophs, *Pseudanabaena* and *Scytonema*, were also detected in each sample, but occupied only very small percentage except in SC where they comprised ∼25% of the total diazotrophs. There were in general very few δ*-Proteobacteria* (i.e., just ∼6–14%) in all samples. In this class, the highest abundance of *Pelobacter* and *Geoalkalibacter* was detected in the SC (11%) and PS (4%), respectively, whereas similar amounts of *Geobacter* were revealed in SM (5%) and SJ (4%). With regards to the α*-Proteobacteria* class, these were in the highest abundance in SM (16%), and they were comprised mainly of *Azospirillum*, *Bradyrhizobium*, and *Rhodoplanes*. More OTUs related to Cluster II were revealed based on the phylogenetic relationship of the 100 most abundant OTUs and distributions of the three *nifH* clusters varied among samples (Supplementary Figure S3). Cluster I was dominant in both SM and SC, such that the latter was almost entirely composed of Cluster I OTUs, with very few Cluster III and no Cluster II OTUs at all being present in this location. Within Cluster I, γ*-Proteobacteria* was the major class among all the samples. In addition, more groups of Cluster II were recovered from PRP (18.97%), which is in agreement with the abundance distribution pattern revealed in the phylogenetic tree (**Figure 4**).

### Comparison among the Different Locations

**FIGURE 6:**
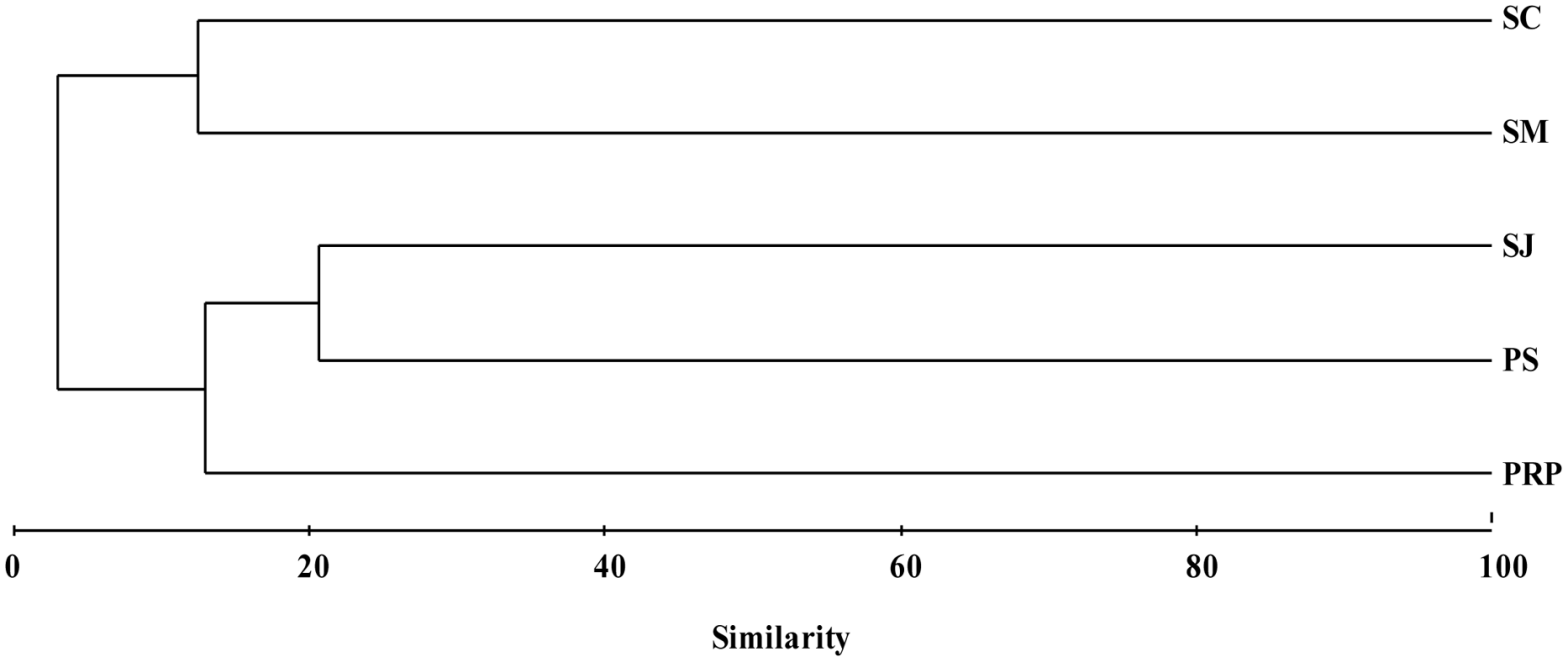
**Unweighted pair group method with arithmetic mean clustering of the diazotrophic community structures for samples collected from the five locations in Singapore based on the total OTUs.** Data were square root transformed and the Bray–Curtis similarity was used for clustering analysis.

Unweighted pair group method with arithmetic mean clustering based on the total OTUs detected in all the samples demonstrated a clear shift among the different geographic locations. SJ and PS had a similar diazotrophic community composition and formed a separate cluster, which was distinct from that in SM and SC (**Figure 6**). This is in agreement with the fact that SJ and PS were mainly comprised of Cluster III (*Desulfovibrio*), while there were more γ*-Proteobacteria* in SM (*Pseudomonas* sp.) and SC (*Marinobacterium*) (**Figure 5**).

**FIGURE 7:**
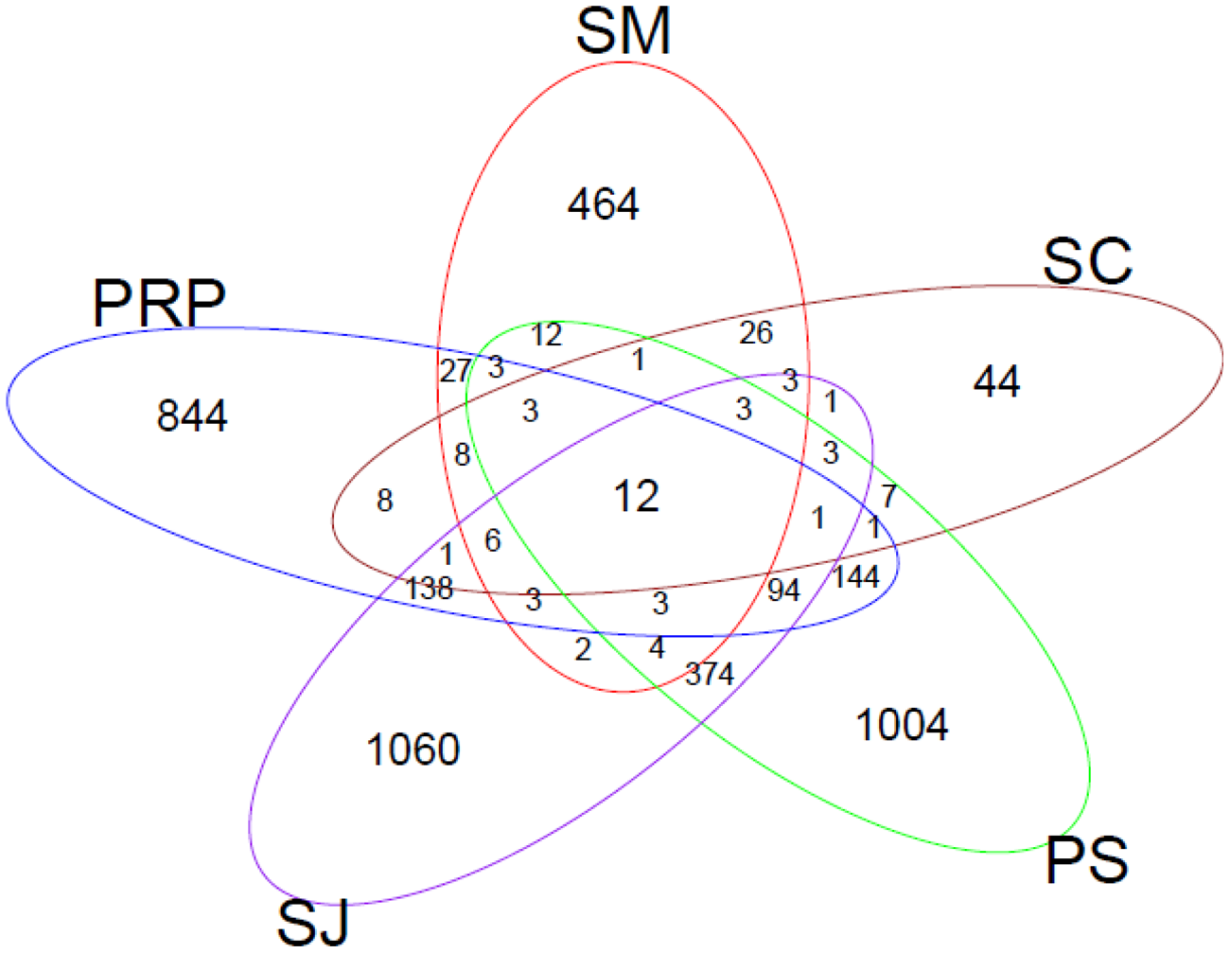
**Venn diagrams representing the overlap of OTUs (with 95% similarity as the cutoff value) for samples collected from the five locations in Singapore**.

Venn diagrams were plotted to show the similarities in terms of the overlapping of OTUs (5% sequence cutoff value) among the sampling locations (**Figure 7**). In total, 12 OTUs (with different similarities) were shared by all the samples. These were: *Marinobacterium lutimaris* (OTU 1, 11, 40, and 88); *Pseudomonas stutzeri* (OTU 23 and 28); *Desulfovibrio aespoeensis* (OTU 307); *Desulfovibrio vulgaris* (OTU83); *Cytonema* sp. (OTU 2); *Pelobacter carbinolicus* (OTU 16); *Azorhizobium caulinodans* (OTU 52); and nitrogen-fixing bacterium TS210 (OTU170). The highest number of common OTUs was found in SJ and PS; and both of these locations shared more OTUs with PRP than with SC and SM. In terms of specific OTUs, SJ had the highest number (i.e., 1060 OTUs), when compared with just 44 in SC.

**FIGURE 8:**
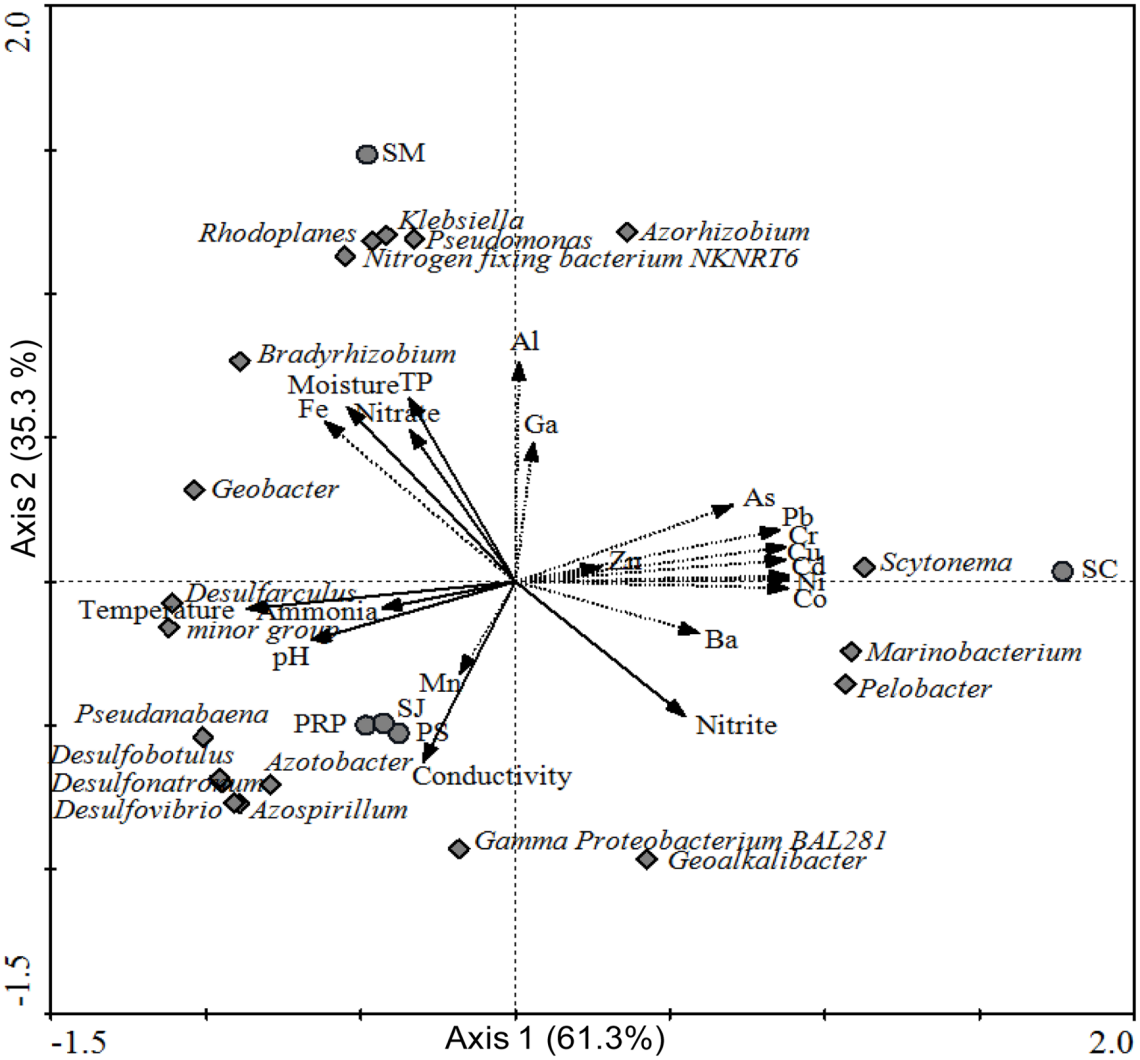
**A redundancy analysis (RDA) biplot based on phylogenetic groups at the genus level for samples collected from the five different locations, with environmental factors as explanatory variables**.

Furthermore, multivariate analysis was performed to show the relationship between diazotrophic community structures recovered from different locations and the associated environmental factors. Axis 1 and 2 of the RDA biplot were shown to contribute 61.3% and 35.3%, to the overall pattern, respectively (**Figure 8**). In addition, the biplot showed that SJ, PS, and PRP had a higher salinity and lower moisture, and they were located close to each other on the lower left panel. In addition, they contained more Cluster III diazotrophs. On the other hand, SC and SM were contaminated with higher concentrations of metals and they were thus located respectively on the upper right and upper left panels. SC was distributed in the direction of As, Pb, Cr, Cu, Cd, Ni, Co, and Ba and close to the *Marinobacterium*, *Scytonema*, and *Pelobacter* genera, whereas SM was closely associated with β/γ*-Proteobacteria* sp., such as *Klebsiella* and *Pseudomonas*.

## Discussion

Microbes play essential roles in the functioning and maintenance of the ecosystem, especially in nitrogen cycling. Nitrogen fixation, which is carried out by nitrogen-fixing bacteria and archaea, is fundamentally important for relieving the nitrogen limitation constraints of an ecosystem. Mangroves are unique and diverse coastal ecosystems that are commonly nitrogen limited. In recent years, the diazotrophs inhabiting the mangrove rhizosphere have been assessed using classical cultivation approaches (Rameshkumar and Nair, 2009) and clone libraries (Flores-Mireles et al., 2007). However, both of these methods demonstrated a far lower diversity of diazotrophs than we showed in our study using high-throughput pyrosequencing of the functional *nifH* gene. In another pyrosequencing study in which mangrove rhizosphere bacteria were characterized, a large number of *Proteobacteria* that were potentially associated with the sulfur cycle were identified, but only a few OTUs belonging to diazotrophic *Proteobacteria* were described (Kersters et al., 2006). Because *Proteobacteria* is a highly diverse and active group in biogeochemical cycling, it is hard to confirm the role these microbes play in a natural community based on the 16S rRNA genes alone. Our study demonstrated that pyrosequencing of the functional *nifH* gene is preferable for a detailed study of diazotrophs living in different environments including in the mangrove rhizosphere.

### Diazotroph Diversity

The increased diversity of the bacterial community has been assumed to be a buffer against the effect of environmental variations. Not surprisingly, in our study the greatest diversity and species richness of diazotrophs was found in the pristine SJ. This high genetic diversity might play a role in the diverse metabolic potentials required to optimize the ability of diazotrophs to adapt to altered environmental conditions. On the other hand, a much reduced level of diversity and species richness was observed in the heavily polluted locations, i.e., SC and SM. These results support a previous report, which demonstrated that the total microbial functional diversity, including that of *nifH*, was significantly reduced in five oil-contaminated fields when compared with pristine fields, due to the toxicity of the oil contaminants (Liang et al., 2011). Su et al. (2007) also reported that agrochemicals exerted toxic effects on diazotrophs and significantly decreased the *nifH* richness and diversity in soil. These findings suggested that unpolluted locations harbor a high diversity of microbes so as to have a higher buffer capacity. Exterior contaminants might cause a range of complex community changes, usually with a reduced community diversity compared with pristine locations, where the microbial community is in a steady state. Even though the pool of dissolved inorganic nitrogen has previously been suggested to be the strongest agent modulating the activity of nitrogenase (Ghosh et al., 2010), we showed that PRP and PS had high NH_4_^+^ concentrations but still showed reasonable nitrogenase diversity. This might be explained by the fact that our study was based on *nifH* sequences at rDNA level, and therefore did not reflect the activity of nitrogenase *per se*. More importantly, these two locations were dominated by sulfate-reducing bacteria, which are known to be involved in nitrogen fixation and sulfate reduction as well as playing a key role in sedimentary cycling of N, C, and S (Lyimo et al., 2002; Varon-Lopez et al., 2014; Romero et al., 2015).

### Diazotroph Phylogeny

The *nifH* sequences obtained in this study were different from those obtained from rhizospheric isolates, and the long branches in the phylogenetic tree suggest that our sequences are also very different from the reference sequences available in GenBank, and so they may therefore represent novel phylotypes. In our study, for comparison purposes, we treated the pristine SJ as our background and attributed the differences in the diazotrophic communities observed in the other mangroves to anthropogenic activities. However, since no historical data are available for our various study locations, it is hard to propose that any particular genus and species are new or endemic to these different ecosystems.

Consistent with previous reports that describe the major diazotroph groups in different natural environments (Zehr et al., 2003; Flores-Mireles et al., 2007), cluster I and III diazotrophs predominated in all five of our study locations. Cluster I consist of the conventional Mo-containing *nifH*, along with some *vnfH*, which was represented by the widely distributed assemblage of *Proteobacteira* and *Cyanobacteria*. Cluster III are usually retrieved from anaerobic environments or anaerobic microsites and can fix nitrogen in anoxic ammonium-rich waters (Farnelid et al., 2013). Cluster II diazotrophs were also detected in our study but they were much less abundant than clusters I and III. This supports the notion that cluster II, which contain *anfH* and nitrogenases from some archaea, in general have a low presence in nature (Zehr et al., 2003). Cluster IV diazotrophs was not recovered from our study but have been reported previously (Flores-Mireles et al., 2007). This discrepancy might be due to the fact that in the earlier report, root samples rather than sediments were used for DNA extraction and different *nifH* primers were also utilized (Flores-Mireles et al., 2007).

As an important player in nutrient cycling, *Proteobacteria* are metabolically highly diverse and widely distributed in various environments (Kersters et al., 2006). The α-, β, γ, and δ-subdivisions of *Proteobacteria* are commonly the major diazotrophic groups in mangrove sediment (Zhang et al., 2008; Andreote et al., 2012). This is in contrast to the symbiotic nitrogen-fixers that are primarily present in agricultural soils (Peoples et al., 1995). In the tropical mangrove, roots display a high production of dry biomass of about 28 tons per hectare per year (Vogt et al., 1986), which enables them to facilitate the development of certain microbial guilds essential for nutrient cycling and ecological resilience. α*-Proteobacteria* are the dominant diazotroph in terrestrial (Smit et al., 2001) environments. This class is comprised of several symbiotic N-fixers such as various *Bradyrhizobium* sp., which are well-known root-nodule bacteria. They are known to be excellent survivors across diverse environmental conditions and have been used as plant inoculants worldwide. It wasn’t surprising, therefore, to find *Bradyrhizobium* in all the locations of our study.

The *Pseudomonas* and *Azotobacter* genera (in the β/γ*-Proteobacteria* class) are common components of rhizosphere diazotrophs (Liu et al., 2012; Shu et al., 2012), and they were also detected in our study. SM contained relatively more *Pseudomonas* and *Klebsiella*. *Klebsiell*a are found routinely as normal flora but they can also act as opportunistic pathogens, and some sub-lineages have developed specific biogeochemical adaptations (Bagley, 1985). γ*-Proteobacteria* nitrogen fixers are also widespread and they have been reported to be the most important heterotrophic diazotrophs in tropical and subtropical oceans (Bird et al., 2005). Our findings reinforce the prominence of *Proteobacteria*, particularly γ*-Proteobacteria*, as the main diazotrophic group in mangrove samples (Dias et al., 2012; Fernandes et al., 2014). In our study, for example, SC contained a high level (i.e., 39.5%) of *Marinobacterium lutimaris. Marinobacterium* is known to degrade various hydrocarbons as its sole carbon and energy source, and has been identified in areas with oil contamination including mangroves (Brito et al., 2006; dos Santos et al., 2011). As the distribution of *Marinobacterium* correlates well with the spatial distribution of petroleum pollutants in mangrove sediments, its abundance might be used to evaluate environmental perturbations. This genus has therefore been suggested to be a useful bioindicator of hydrocarbon pollution in mangrove ecosystems (dos Santos et al., 2011). *Marinobacterium* has the capability to compete with other microorganisms in order to survive for long periods in mangrove sediments (Alfaro-Espinoza and Ullrich, 2015). *Marinobacterium* also exhibits a higher rate of nitrogen-fixation in the presence of mangrove roots, which indicates a possibly beneficial mutualistic interaction between these bacteria and the mangrove plants (Alfaro-Espinoza and Ullrich, 2015).

The SC sampling location also contained a high level of *Scytonema* sp., which belongs to the *Nostocales* (heterocyst-forming) order of cyanobacteria. Another cyanobacterial diazotroph, *Pseudoanabaena*, was found in all our locations with low abundance; these are frequently detected in mangrove ecosystems (Kyaruzi et al., 2003). Moreover, filamentous non-heterocystous cyanobacteria *Microcoleus* were detected at all five sampling sites. *Microcoleus* are known to build microbial mates in a variety of environments (Stal, 2000). It is reported that *Microcoleus chthonoplastes* contains a complete nif-gene cluster, which is believed acquired from *Deltaproteobacteria* through horizontal gene transfer (Bolhuis et al., 2010). *Microcoleus chthonoplastes* has been reported to fix dinitrogen by the temporal separation between photosynthetic O_2_ evolution and N_2_ fixation strategy (Pearson et al., 1981), although active nitrogen fixation has not been observed in pure cultures (Bolhuis et al., 2010). In our study, this species was found more abundant in relative pristine sites of SJ (1.86% of total sequences) and less polluted PS (1.89%) and PRP (1.22%) than in heavily polluted sites of SM (0.51%) and SC (0.19%), suggesting that they are rather sensitive to nutrient and heavy metal pollution.

The cluster III microbes are a diverse group that are wholly comprised of anaerobes, which are distantly related to each other, such as δ*-proteobacteria*, green sulfur bacteria and Archaea. *Proteobacteria* usually form a distinct cluster and archaeal nitrogenases, including homologues of both functional nitrogenases and *nif*-like genes of unknown function, have thus far only been found in methanogens (Chien et al., 2000). In our study, the Cluster III diazotrophs were composed entirely of the sulfate-reducing bacteria, which are very common in lake beds (Bak and Pfennig, 1991) and mangrove sediments (Lyimo et al., 2002; Varon-Lopez et al., 2014; Romero et al., 2015). It has been reported that they are dominant in mangrove sediments that are subjected to long-term fertilization with nitrogen and phosphorus (Romero et al., 2015). They have positive ecological effects on the bioremediations of heavy-metal contaminated soils (Jiang and Fan, 2008) and wetland (Moreau et al., 2013), where they are capable of detoxifying contaminants and degrading complex substrates, such as long-chain and aromatic hydrocarbons (Muyzer and Stams, 2008). Their presence, therefore, indicates the potential of bioremediation and the resiliency of the ecosystem to anthropogenic activities (Heidelberg et al., 2004; Pérez-Jiménez and Kerkhof, 2005).

### Environmental Effects on Diazotrophs

Microbial diversity and activity are fundamental for the productivity and resilience of mangroves (Gomes et al., 2010; dos Santos et al., 2011). Our results match those from previous studies conducted with sediments from pristine (Dias et al., 2010), and urban (Gomes et al., 2008) mangroves, from areas affected or not affected by shrimp farms (Sousa et al., 2006), and in mangrove systems contaminated by oil and industrial contaminants (Tian et al., 2008; Taketani et al., 2010), which indicate that microbes in the mangrove sediments around the world are strongly influenced by biogeochemical, anthropogenic, and ecological events.

Unlike diazotrophs living in the mangrove sediments, which are influenced by the nutrient levels and the types of organic and inorganic compounds in the sediments alone (Ghosh et al., 2010), diazotrophs associated with the mangrove rhizosphere are likely to additionally be affected by root exudates (Glick et al., 1999). However, significant changes in the community of diazotrophs in mangrove sediments induced by long-term fertilization of nitrogen and phosphorus have also been reported (Romero et al., 2012, 2015). In our study, the fact that the highest *nifH* diversity was found in SJ is not surprising as this location has a low ammonia and nitrate content; likewise, the various different diazotroph groups can help to relieve the nitrogen deficiency. However, genetic diversity of *nifH* were reduced or completely removed in locations with high levels of ammonia that were caused by anthropogenic activities. With regard to the reasonably high nitrogenase diversity at PRP and PS despite high ammonium concentrations, it may be explained by the fact that our study is based on DNA level, so that it does not indicate the activity of nitrogenase. Moreover, relative high phosphorus concentrations in studied sites provide favorable condition for nitrogen fixation. The diazotroph communities in these two locations were dominated by sulfate-reducing bacteria, which are involved in the microbial processes of nitrogen fixation and sulfate reduction and play a key role in sedimentary cycling of N, C, and S (Lyimo et al., 2002; Varon-Lopez et al., 2014), but the control mechanisms for their nitrogenase activity remain unsolved.

Metal exposure also leads to the establishment of tolerant microbial populations. For example, reduced nitrogenase activity has been reported previously in heavy metal contaminated soil treated with sewage sludge (Lorenz et al., 1992). In our study, two locations were affected by extreme anthropogenic activity, SM was affected by intense agricultural activities, and SC was affected by an old landfill and the adjacent airport. Both of these locations contained high concentrations of metals and nutrients, which resulted in a significantly altered diazotrophic community. Significantly high abundance of *Marinobacterium* sp. was detected in SC, this group is known to occur in areas of pollutant degradation and mangrove remediation. The waters near to PRP had previously (i.e., a couple of years prior to our sampling) been affected by toxic algal blooms. The relatively high nutrient levels measured in this location compared with those of SJ, can therefore be explained by the intermediate community changes brought about by the rhizosphere diazotrophs. Regarding the two landfill sampling sites, even though the old site (at SC) was closed in 1999, the high content of heavy metals detected in our samples suggests that the surrounding natural environment was still being affected. The heavy metals thus had a long-term effect on the adjacent areas by inducing a change in the local microbial communities. In the new landfill site (at PS), the concentration of heavy metals was not very high in the sediment, but the concentration of nutrients was significantly greater than those measured in the pristine location at SJ. These results might therefore act as a warning signal to the government, as they clearly indicate the long-term negative impact of landfills on the environment.

## Conclusion

Our study represents the first application of high-throughput pyrosequencing on the functional *nifH* gene to investigate the anthropogenic impact on diazotrophs of the mangrove rhizosphere. We demonstrated marked differences in the diazotrophic community structure in conjunction with reduced species diversities in various heavily polluted locations when compared with a pristine location. The abundance of certain genera that are known bioindicators of pollution, as well as those with bioremediation potential, suggest that anthropogenic activity had a negative impact on the diazotroph diversity in the mangrove rhizosphere. Therefore, proper policies and measures need to be established to alleviate the pollution of mangroves, and to sustain the high activity of nitrogen fixation and maintain the overall health of these ecosystems. Considering the different nitrogenase homologues assembled with different *nif* gene arrangements (Dominic et al., 2000), our study, which was based purely on the *nifH* gene, might have resulted in an underestimation of diazotroph diversity. Future studies that include other *nif* genes (based on the level of RNA expression reflecting the nitrogen-fixing activities) together with *in situ* nitrogen-fixing rate measurements will help us to obtain a more comprehensive understanding of the link between the diversity of functional genes and the biogeochemical activities of diazotrophs in this ecosystem. They will also allow a more precise estimation of the impact of anthropogenic and environmental changes on the ecological function of mangrove ecosystems.

## Conflict of Interest Statement

The authors declare that the research was conducted in the absence of any commercial or financial relationships that could be construed as a potential conflict of interest.

## Footnotes

1 http://fungene.cme.msu.edu/FunGenePipeline/framebot/form.spr

2 http://www.ncbi.nlm.nih.gov/
