# Supplementary material for: Anthropogenic impact on diazotrophic diversity in the mangrove rhizosphere revealed by nifH pyrosequencing
Source: Front Microbiol. 2015 Oct 21;6:1172. doi: 10.3389/fmicb.2015.01172 (PMC4612719; doi:10.3389/fmicb.2015.01172)
Supplement: Supplementary file 1 [file Data_Sheet_1.PDF]

Supplementary materials

Figure S1. Rarefaction curves for *nifH* gene sequences obtained by amplicon pyrosequencing from samples collected in five locations in Singapore with 95% similarity as cutoff value.

Figure S2. Relative abundance and affiliation of the 10 most abundant OTUs in samples collected from five locations in Singapore.

Figure S3. Relative abundance of each diazotrophic cluster based on the classification of the most abundant 100 OTUs in samples collected from five locations in Singapore.

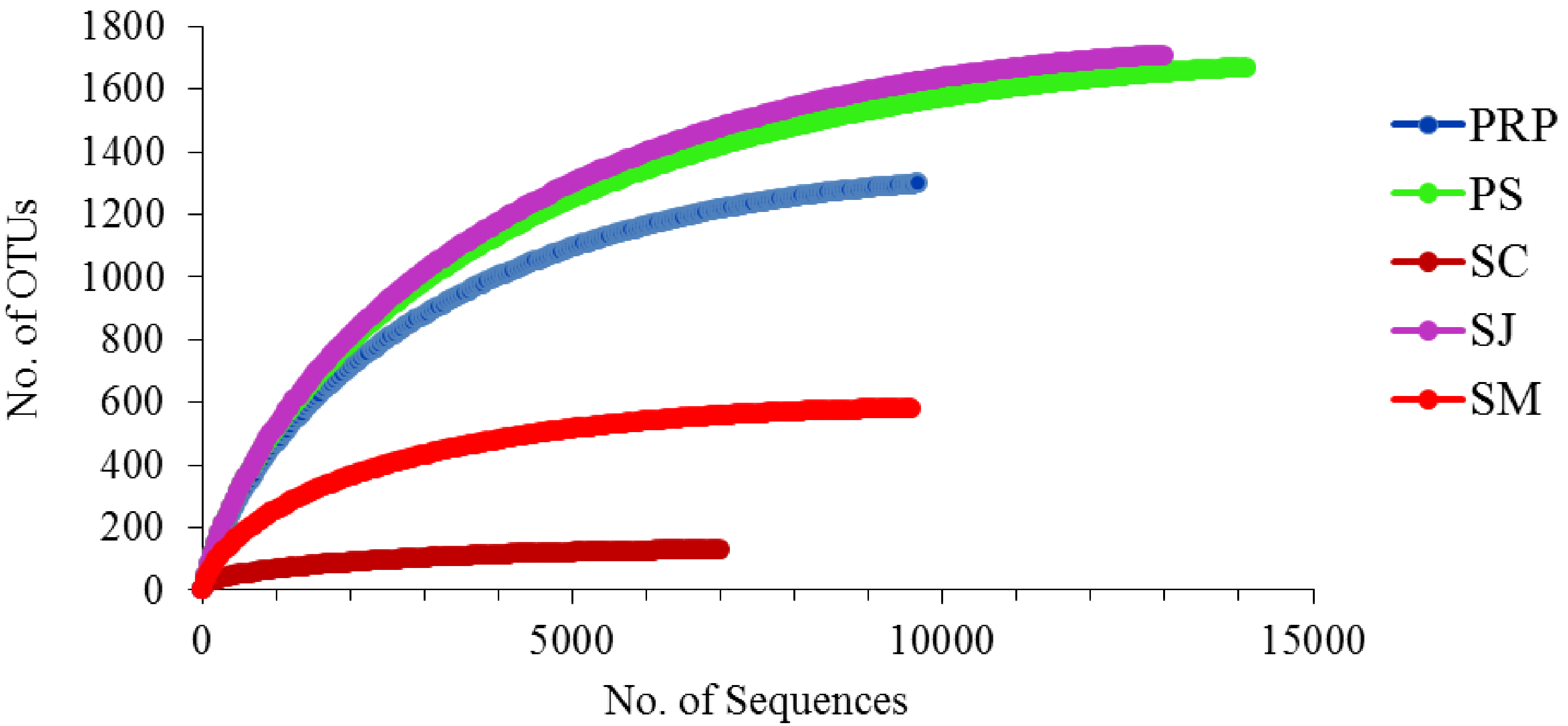

No. of OTUs
1800
1600
1400
1200
1000
800
600
400
200
0
5000
10000
15000
No. of Sequences
PRP
PS
SC
SJ
SM

Figure S1

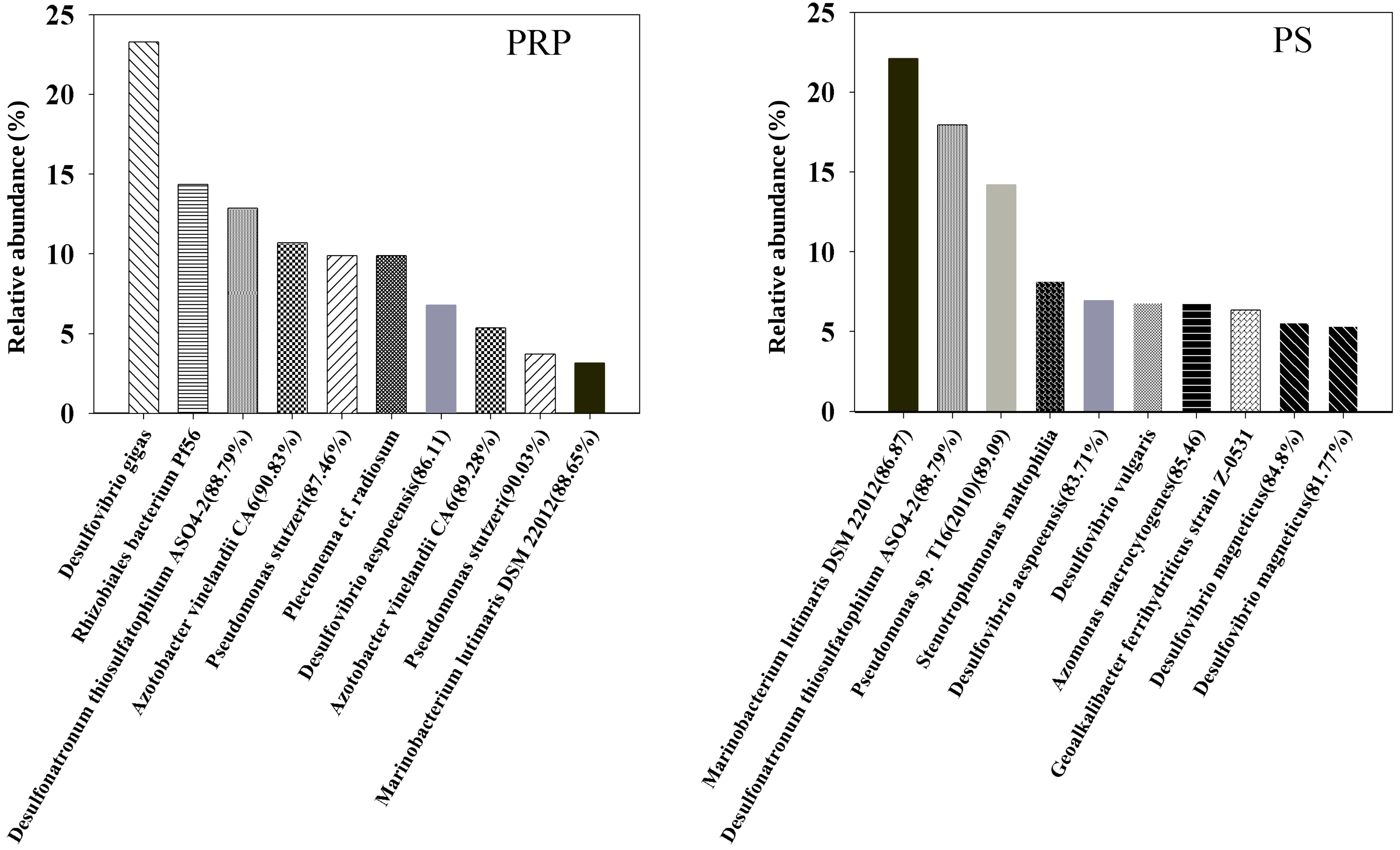
PRP
Relative abundance (%)
0
5
10
15
20
25
Desulfovibrio gigas
Rhizobiales bacterium Pf56
Desulfonatronum thiosulfatophilum ASO4-2(88.79%)
Azotobacter vinelandii CA6(90.83%)
Pseudomonas stutzeri(87.46%)
Plectonema cf. radiosum
Desulfovibrio aespoeensis(86.11)
Azotobacter vinelandii CA6(89.28%)
Pseudomonas stutzeri(90.03%)
Marinobacterium lutimaris DSM 22012(88.65%)
PS
Relative abundance (%)
0
5
10
15
20
25
Marinobacterium lutimaris DSM 22012(86.87)
Desulfonatronum thiosulfatophilum ASO4-2(88.79%)
Pseudomonas sp. T16(2010)(89.09)
Stenotrophomonas maltophilia
Desulfovibrio aespoeensis(83.71%)
Desulfovibrio vulgaris
Azomonas macrocytogenes(85.46)
Geoalkalibacter ferrihydriticus strain Z-0531
Desulfovibrio magneticus(84.8%)
Desulfovibrio magneticus(81.77%)

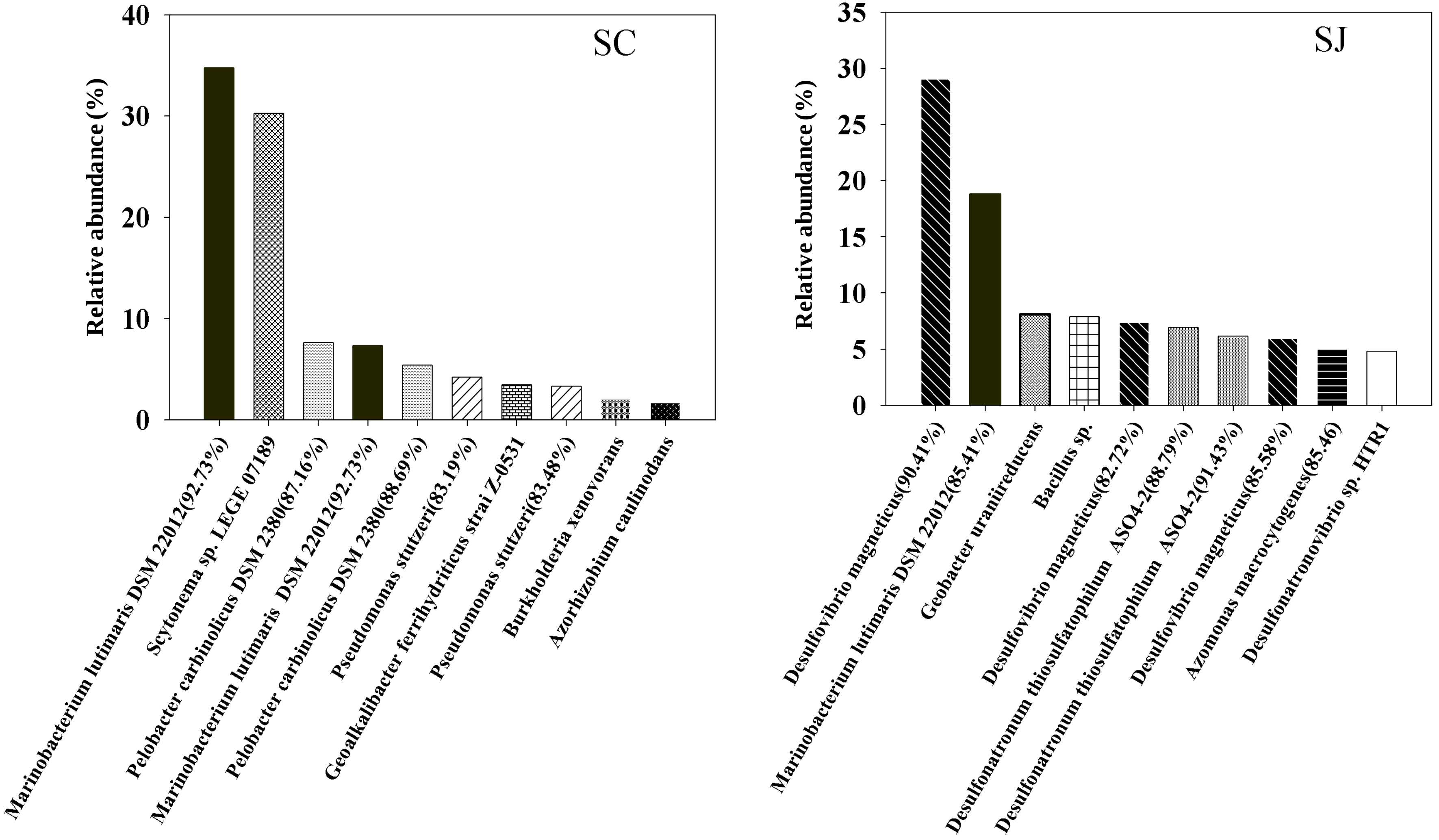
SC
Relative abundance (%)
0
10
20
30
40
Marinobacterium lutimaris DSM 22012(92.73%)
Scytonema sp. LEGE 07189
Pelobacter carbinolicus DSM 2380(87.16%)
Marinobacterium lutimaris DSM 22012(92.73%)
Pelobacter carbinolicus DSM 2380(88.69%)
Pseudomonas stutzeri(83.19%)
Geoalkalibacter ferrihydriticus strai Z-0531
Pseudomonas stutzeri(83.48%)
Burkholderia xenovorans
Azorhizobium caulinodans
SJ
Relative abundance (%)
0
5
10
15
20
25
30
35
Desulfovibrio magneticus(90.41%)
Marinobacterium lutimaris DSM 22012(85.41%)
Geobacter uraniireducens
Bacillus sp.
Desulfovibrio magneticus(82.72%)
Desulfonatronum thiosulfatophilum ASO4-2(88.79%)
Desulfonatronum thiosulfatophilum ASO4-2(91.43%)
Desulfovibrio magneticus(85.58%)
Azomonas macrocytogenes(85.46)
Desulfonatronovibrio sp. HTR1

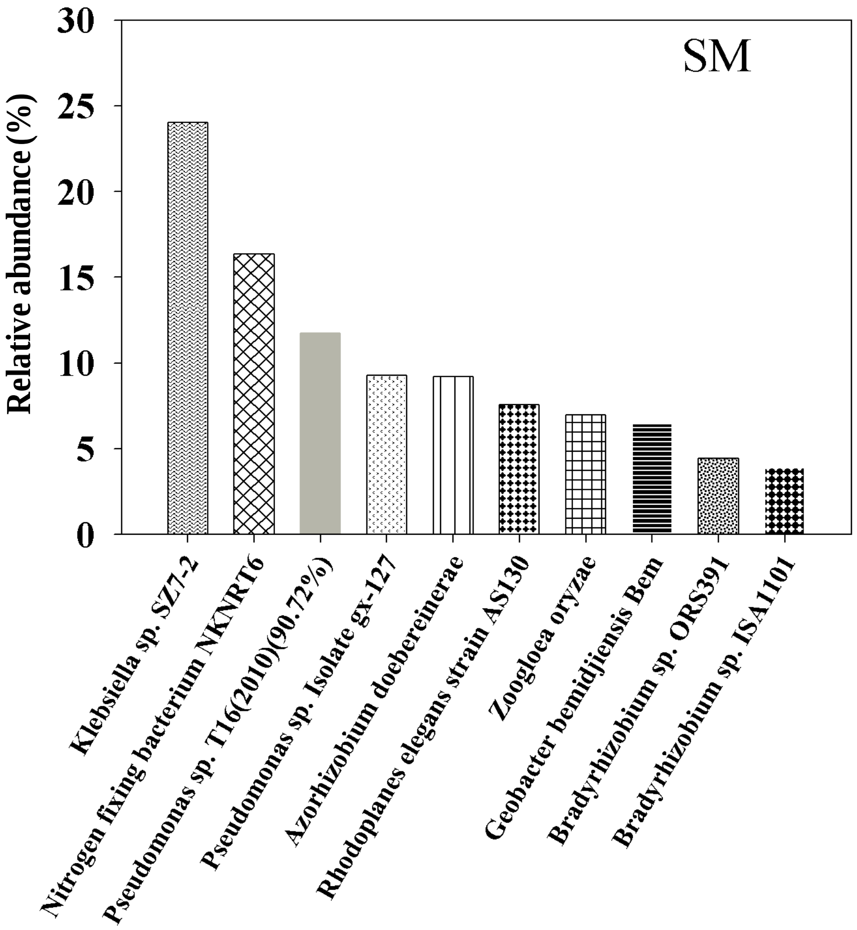
SM
Relative abundance (%)
0
5
10
15
20
25
30
Klebsiella sp. SZ7-2
Nitrogen fixing bacterium NKNRT6
Pseudomonas sp. T16(2010)(90.72%)
Pseudomonas sp. Isolate gx-127
Azorhizobium doebereinerae
Rhodoplanes elegans strain AS130
Zoogloea oryzae
Geobacter bemidjiensis Bem
Bradyrhizobium sp. ORS391
Bradyrhizobium sp. ISA1101

Figure S2

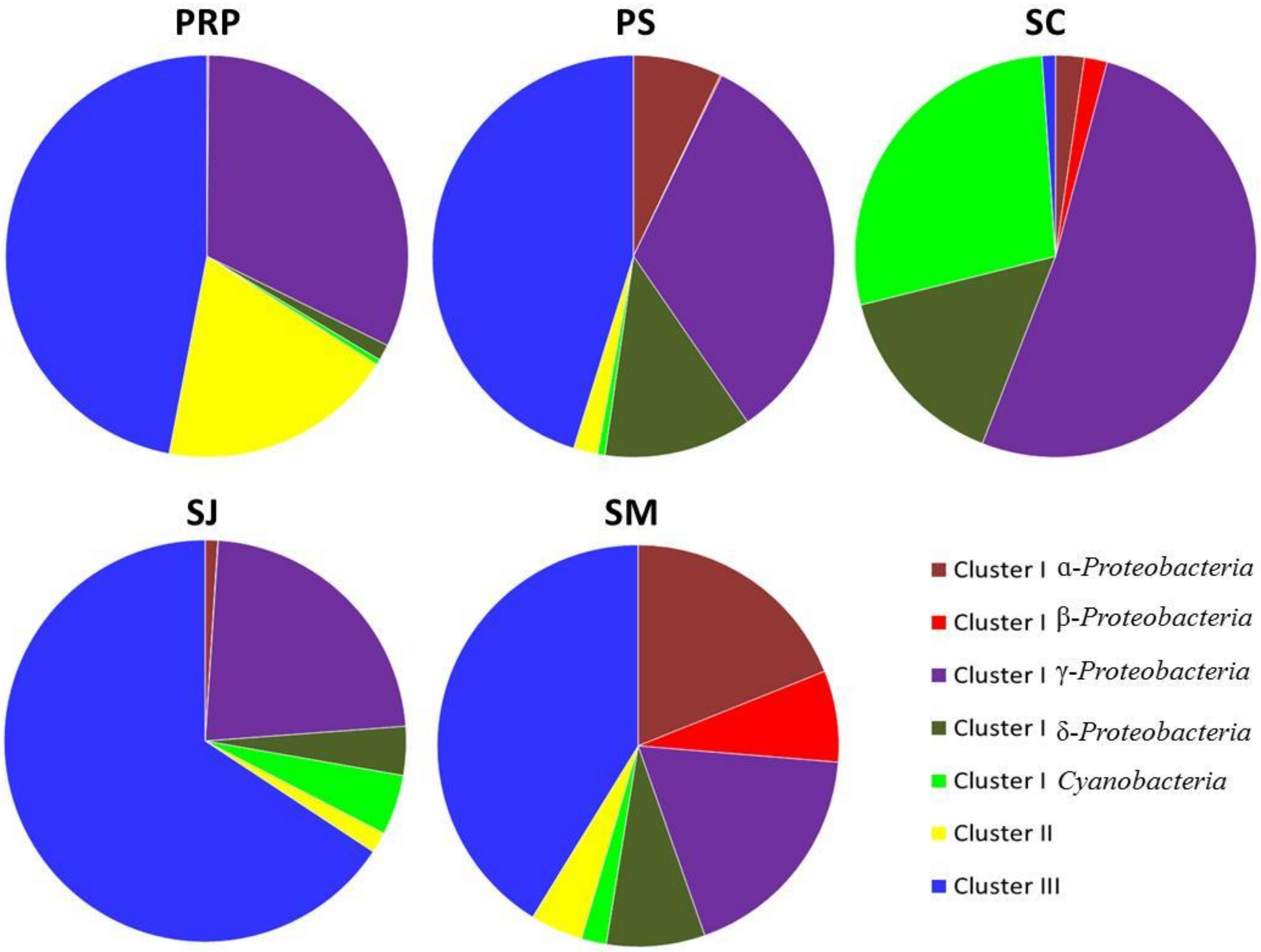
PRP
PS
SC
SJ
SM
Cluster I α-*Proteobacteria*
Cluster I β-*Proteobacteria*
Cluster I γ-*Proteobacteria*
Cluster I δ-*Proteobacteria*
Cluster I *Cyanobacteria*
Cluster II
Cluster III

Figure S3
